# Impaired Global Precedence Effect in Severe Alcohol Use Disorder and Korsakoff’s Syndrome: A Pilot Exploration through a Global/Local Visual Paradigm

**DOI:** 10.3390/jcm12113655

**Published:** 2023-05-25

**Authors:** Anne Lise Pitel, Alice Laniepce, Céline Boudehent, Nicolas Poirel

**Affiliations:** 1 Normandie Université, UNICAEN, INSERM, U1237, PhIND “Physiopathology and Imaging of Neurological Disorders”, Institut Blood and Brain @ Caen-Normandie, Cyceron, 14074 Caen, France; alice.laniepce@univ-rouen.fr (A.L.);; 2 Normandie Université, UNIROUEN, CRFDP (EA 7475), 76821 Rouen, France; 3 Université Paris Cité, LaPsyDÉ, CNRS, 75005 Paris, France; nicolas.poirel@u-paris.fr; 4 GIP Cyceron, 14000 Caen, France

**Keywords:** local–global processing, compound stimuli, global precedence effect, identification processing

## Abstract

In healthy populations, visual abilities are characterized by a faster and more efficient processing of global features in a stimulus compared to local ones. This phenomenon is known as the global precedence effect (GPE), which is demonstrated by (1) a global advantage, resulting in faster response times for global features than local features and (2) interference from global distractors during the identification of local targets, but not vice versa. This GPE is essential for adapting visual processing in everyday life (e.g., extracting useful information from complex scenes). We investigated how the GPE is affected in patients with Korsakoff’s syndrome (KS) compared to patients with severe alcohol use disorder (sAUD). Three groups (including healthy controls, patients with KS and patients with sAUD) completed a global/local visual task in which predefined targets appeared at the global or local level during either congruent or incongruent (i.e., interference) situations. The results showed that healthy controls (N = 41) presented a classical GPE, while patients with sAUD (N = 16) presented neither a global advantage nor global interference effects. Patients with KS (N = 7) presented no global advantage and an inversion of the interference effect, characterized by strong interference from local information during global processing. The absence of the GPE in sAUD and the interference from local information in KS have implications in daily-life situations, providing preliminary data for a better understanding of how these patients perceive their visual world.

## 1. Introduction

Korsakoff’s syndrome (KS) is a major neurocognitive disorder resulting from thiamine deficiency and is mainly characterized by severe, persistent and debilitating amnesia. It is most frequently observed in patients with severe Alcohol Use Disorder (sAUD), who are particularly at risk of thiamine deficiency due to poor diet, drinking habits and altered thiamine absorption from the gastrointestinal tract [1]. Most cognitive studies on KS have focused on high-level functions, particularly memory [2,3] and executive abilities [4,5]. Conversely, the investigation of lower-level processing stages, particularly visuoperceptive abilities, in KS is scarce. This is surprising because such deficits are frequently reported in sAUD, are considered as persistent over time and potentially underlie higher-level cognitive impairments (see [6] for a review).

The limited literature suggests the existence of visuoperceptive impairments in KS. Indeed, patients with KS show reduced performance on the Digit Symbol test (but not on other subtests of the WAIS-R involving visuoperceptive processing), with KS being associated with more severe impairments than sAUD [7]. These results confirm earlier findings showing reduced visuospatial abilities in KS compared to healthy controls (HC) and/or sAUD patients [8]. However, this deteriorated performance might be partly explained by a reduced processing speed rather than visuoperceptive difficulties. More recently, Kasse et al. [9] reported impaired object perception (with preserved spatial perception) in KS patients compared to healthy controls, but without comparison with an sAUD group. To sum up, previous studies have systematically reported altered visuoperceptive processes in KS, but two major questions remain unanswered, namely (1) the specificity of the impairment compared to that observed in sAUD and (2) the generalizability of this deficit to other visuoperceptive processes identified as essential for adapted everyday functioning.

A key visuoperceptive ability yet to be explored in KS is global/local processing, which is required on a daily basis to perceive a visual scene with different hierarchical levels of structure, from the largest global level of organization to the smallest local elements. For example, the perception of a forest is based on a global analysis, whereas a local analysis is necessary to detect the individual objects in the scene (i.e., the trees) or the distinct features of each object (e.g., leaves and branches). To study the mechanisms that underlie global and local perception, Navon used compound stimuli consisting of large letters (the global level) composed of a suitable arrangement of small letters (the local level) [10]. In most paradigms that used compound stimuli, participants had to attend to one level (i.e., the “target” level: global or local) and decide on each trial if a target letter (e.g., “H”) was present in that prespecified level (e.g., the global), while ignoring the other “irrelevant” level (e.g., the small local letters “N”, the arrangement of which forms the global “H”). Such paradigms allow to determine which level (global/local) is processed faster, but also the interference effect of the irrelevant level on the targeted one. Two well-established effects have been documented in healthy populations: a global advantage (global processing is faster than local one) and a global interference (global processing interferes with local one) (reviewed in [11], see also [12]). These effects are called the “Global Precedence Effect” (GPE).

Previous investigations of the GPE in sAUD have yielded contradictory results. The Navon paradigm initially showed a preserved processing speed and attentional allocation, but higher error rates in patients compared to controls. Qualitatively, the GPE was similar in sAUD patients and controls, but quantitatively, patients with sAUD showed a larger interference effect from global information during local processing [13]. However, the reverse pattern of results emerged in a more recent brain behavior study [14]. Although patients with sAUD were as accurate as the controls, they were slower and showed an interference effect of local processing on global processing (i.e., a local precedence effect). This stronger impairment of global processing compared to local processing confirms earlier findings in sAUD [15,16]. This contradiction between the results of Wegner et al. [13] and Müller-Oehring et al. [14] may be due to differences in attentional demands; during the global–local task performed by Wegner et al. [13], participants had to perform a divided attention task which required them to consider both global and local information in each trial and evaluate switching abilities [17]. Conversely, Müller-Oehring et al. [14] employed a focused attention task, where participants processed either global or local information in each trial. This task allowed for the investigation of global and local processes individually (e.g., [12,18]), as well as the consideration of global and local interference effects [18].

While these results suggest modifications in the GPE in sAUD, this visuoperceptive component remains unexplored in KS. Therefore, we investigated local–global processing in KS and compared it with sAUD using a classical global–local paradigm. We compared HC, patients with sAUD and patients with KS. Following previous findings [6], we hypothesized a gradient of altered GPE, where healthy controls would show an expected GPE, patients with sAUD would present an altered GPE and patients with KS would present an even more altered GPE than patients with sAUD. Our paradigm will not only allow us to investigate fine-grained local and global visuoperceptive processes but also to explore local and global interference effects.

## 2. Materials and Methods

### 2.1. Population

We enrolled 7 patients with KS, 16 patients with sAUD and 41 healthy controls (HC). We matched the groups in terms of education (*F*(2, 61) = 1.03, *p* = 0.381) but not in terms of gender (X^2^ = 22.2, *p* < 0.001) or age (*F*(2, 61) = 9.24, *p* = 0.001). There were only men among patients with sAUD, only women among patients with KS and both genders among HC. Patients with KS were older than patients with sAUD (*p* = 0.03) and HC (*p* = 0.001), while there was no significant difference between sAUD and HC (*p* = 0.90, Table 1).

We recruited patients with KS in a nursing home (Maison Vauban, Roubaix, France). They all met the criteria for amnestic-confabulatory-type persistent alcohol-induced major neurocognitive disorder (DSM-5 [19]). They were long-term abstainers but had an AUD history for >20 years (even though we could not collect precise information of their alcohol consumption because of amnesia). All patients’ information was collected through family members and medical charts. Each patient was included after a careful codified selection procedure involving experts in cognitive neuropsychology and behavioral neurology. A detailed neuropsychological examination was conducted to confirm the diagnosis of KS. All patients with KS presented severe episodic memory impairments potentially associated with other cognitive deficits. The French version of the California Verbal Learning Test (CVLT [20]) was used to measure the verbal episodic memory in KS (Table 1). The severity of memory disorders was such that they resulted in severe disabilities in everyday life. Clinical and neuroimaging examinations enabled us to exclude other possible etiologies for memory disorders (including focal brain damage).

Clinicians recruited patients with sAUD while they were receiving withdrawal treatment as inpatients at Caen University Hospital. Patients met the DSM-5 criteria for sAUD [19] for at least 5 years. They were interviewed using a modified version of the semi-structured lifetime drinking history and the Alcohol Use Disorders Identification Test (AUDIT [21]). Measures included daily alcohol consumption during the month before admission (in standard drinks, with a standard drink corresponding to 10 g of pure ethanol) and the duration of abstinence at inclusion (Table 1). Although patients were in early abstinence, none of them presented with physical symptoms of alcohol withdrawal, as assessed by the Cushman’s scale at inclusion [22].

We administered the AUDIT questionnaire to the HC to ensure that they did not meet the criteria for hazardous drinking (AUDIT < 7 for men and <6 for women [21]).

All participants spoke French as their native language and did not have previous history of neurological or psychiatric disorders or severe brain injury (except for brain abnormalities associated with sAUD and KS). The absence of these pathologies was verified based on a clinical interview of all participants, as well as their medical chart and a magnetic resonance imaging scan of all patients.

Clinical neuroimaging examinations were performed on patients to rule out other etiologies that could explain memory impairments. All participants (and caregivers for patients when appropriate) provided written informed consent to be included in the study, which was approved by the local ethics committee of Caen University Hospital in accordance with the principles of the Declaration of Helsinki.

We performed an a priori power analysis using G*Power 3.1 [23] with a mixed 3 × 2 × 2 design with the group as the between-subject factor (HC vs. sAUD vs. KS) and the target level (global vs. local) and the trial type (congruent vs. incongruent) as within-subject factors. The analysis indicated that a sample size of 24 participants (8 per group) would be sufficient to detect a medium effect size (f = 0.30) with a power of 0.80 and an alpha level of 0.05.

### 2.2. Apparatus and Procedure

**Figure 1:**
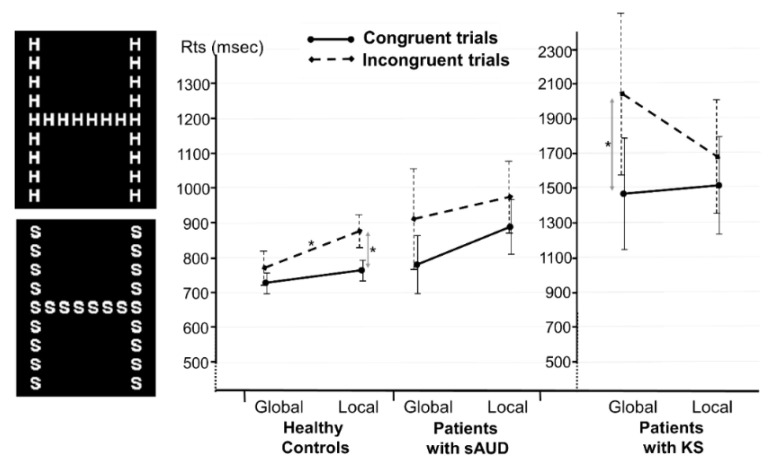
Examples of compound stimuli (**left** panel). Interaction between the group, target level and trial type (**right** panel). * *p* < 0.05. sAUD: severe Alcohol Use Disorder; KS: Korsakoff’s syndrome.

The participants completed a global–local task on a laptop computer using E-Prime software. They were seated approximately 60 cm away from the screen and a psychologist was present throughout the experiment. The participants were initially instructed to focus their attention on either the global or local level, while ignoring the other level. Each trial required the participants to indicate which of the two target letters (H or S) was present at the attended level, while ignoring the other level. The study included two types of trials: congruent trials, where the same letter appeared at global and local levels (Figure 1, upper left part), and incongruent trials, where different possible targets appeared at the two levels (Figure 1, lower left). Each trial began with a fixation cross displayed at the center of the screen for 1500 ms, followed by the compound stimulus that remained visible until the participant responded. After the response, a blank screen was presented for a duration ranging from 500 to 1500 ms. Response times (RTs) and accuracy were recorded. The experiment included 40 trials, comprising 10 congruent global trials, 10 incongruent global trials, 10 congruent local trials and 10 incongruent local trials. The participants completed two blocks of 20 trials, one for the global level and one for the local level. The order of local and global blocks, as well as the order of trials within each block, were randomized. Participants completed four practice trials before the experiment and received feedback on their accuracy.

### 2.3. Statistical Analyses

Participant responses were highly accurate, indicating a ceiling effect (the mean accuracies for the global congruent condition were 99.8% ± 0.1, 98.1% ± 0.5 and 98.6% ± 0.4 for HC, sAUD and KS participants, respectively; for the global incongruent condition, they were 97.3% ± 0.5, 95% ± 1 and 97.1% ± 0.8 for HC, sAUD and KS participants, respectively; for the local congruent condition it was 100% for HC, sAUD and KS participants; and for the local incongruent condition they were 98.1% ± 0.4, 96.9% ± 0.6 and 84.3% ± 2 for HC, sAUD and KS participants, respectively). Only RTs were analyzed using JASP software.

We conducted a three-factor repeated-measure analysis of covariance (ANCOVA) that included RTs for correct responses. The group (HC, sAUD or KS) was used as the between-subject factor, while the target level (global or local) and the trial type (congruent or incongruent) were included as within-subject factors. Age was used as a covariate. We conducted all post hoc comparisons using Bonferroni corrections.

## 3. Results

The repeated-measure ANCOVA on RTs did not show main effects of the target level (*F*(1, 60) = 0.57, *p* = 0.45) or trial type (*F*(1, 60) = 0.04, *p* = 0.84), but it did reveal the significant main effect of the group (*F*(2, 60) = 10.93, *p* < 0.001, η_p_^2^ = 0.27). HC and sAUD patients were faster than KS patients overall (*p* < 0.001) (*F*(2, 60) = 2.05, *p* = 0.14 and *F*(1, 60) = 0.77, *p* = 0.38, respectively). However, there was a significant interaction effect between trial type and group (*F*(2, 60) = 6.62, *p* = 0.003, η_p_^2^ = 0.18), with a trial type effect observed for HC (*p* = 0.03) and KS participants (*p* < 0.001), but not for sAUD participants (*p* = 0.15). While there was a main effect of age (*F*(1, 60) = 6.37, *p* = 0.01), age did not interact with the target level (*F*(1, 60) = 0.6, *p* = 0.44) or trial type (*F* (1, 60) = 3.15, *p* = 0.08), and there was no age × target level × trial type interaction effect (*F*(1, 60) = 0.0003, *p* = 0.99). Finally, there was a significant three-way interaction between target level × trial type × group (*F*(2, 60) = 10.81, *p* < 0.001, η_p_^2^ = 0.27).

As shown in Figure 1, a repeated-measure ANOVA for HC revealed the main effects of target level and trial type (*F*(1, 40) = 6.72, *p = 0*.01, η_p_^2^ = 0.14 and *F*(1, 40) = 35.25, *p* < 0.001, η_p_^2^ = 0.47, respectively), and a target level × trial type interaction effect (*F*(1, 40) = 5.24, *p =* 0.03, η_p_^2^ = 0.12). The interaction was characterized by faster RTs for global incongruent trials compared to congruent trials (774 ± 49 ms and 876 ± 44 ms, respectively, *p* = 0.008) and faster RTs during local congruent trials than during local incongruent trials (765 ± 38 ms and 876 ± 44 ms, respectively, *p* < 0.001), suggesting a GPE. Conversely, the repeated-measure ANOVA for the sAUD group did not show any significant effects of target level (*F*(1, 15) = 1.43, *p* = 0.25) or trial type (*F*(1, 15) = 3.44, *p* = 0.08), and there was no significant target level × trial type interaction (*F*(1, 40) = 0.32, *p* = 0.58), suggesting an absence of a GPE in this group. Finally, the repeated-measure ANOVA for the KS group showed no main effect of target level (*F*(1, 6) = 0.81, *p* = 0.40), a main effect of trial type (*F*(1, 6) = 9.58, *p* = 0.02, η_p_^2^ = 0.65) and a significant target level × trial type interaction effect (*F*(1, 6) = 7.30, *p* = 0.04, η_p_^2^ = 0.55). This interaction was characterized by slower RTs during the global incongruent condition than during the global congruent condition (2037 ± 466 ms and 1467 ± 338 ms, respectively, *p* = 0.01), suggesting a strong interference from local information during global processing in this group.

## 4. Discussion

Using a classical global–local paradigm, we observed the expected GPE effect in healthy controls but an absence of this effect in individuals with sAUD and a reverse pattern of performance with local interference in patients with KS. These findings are important since efficient visuoperceptive processes are essential for adapted everyday life behavior. Human beings need to continuously monitor their surrounding world to correctly perceive, interpret, predict and react to environmental and social stimuli [24], which is crucial for efficient cognitive functions such as attention, memory and executive abilities [25]. Despite their role in higher-level cognition, visual abilities have been poorly investigated in alcohol use disorders, particularly in KS. The available results are sparse, often contradictory and mostly based on general tasks that hinder the exploration of specific processes. Therefore, we aimed to explore a well-established visuoperceptive effect, the GPE, in patients with sAUD or KS using a sound methodological approach. The GPE, characterized by global advantage (faster processing of global over local features of a stimulus) and global interference (stronger global interference on local processing), is essential for a wide-range of everyday behaviors, from reading to driving. Previous results regarding the modification of the GPE in sAUD are mixed, and none had explored it in KS. Thus, we explored the GPE in these two populations through the classical local–global paradigm to establish the gradient of impairments across alcohol use disorders.

Our study produced three main findings. First, we successfully replicated the classic GPE effect among healthy controls. They demonstrated the two key features in this effect: (a) a global advantage, as evidenced by faster reaction times for global stimuli than local stimuli in the incongruent condition, indicating faster processing of global stimuli and (b) a global interference, as evidenced by faster reaction times for congruent stimuli than incongruent stimuli in the local condition (but not in the global condition), indicating a stronger impact of global incongruent stimuli on local processing than local processing on global incongruent stimuli. This replication supports earlier research conducted across various populations and experimental designs [26,27,28], indicating that our paradigm was a reliable and valid assessment of this effect. The ceiling effect observed in all groups further confirms that all participants understood the instructions and performed the task accurately.

Secondly, we found a complete absence of the GPE effect in individuals with sAUD, as they did not exhibit global advantage or global interference. This result is consistent with most previous studies that have shown a reduction in or absence of global precedence in sAUD [14,15,16]. As previously mentioned, one study found a contradictory pattern, showing an increased GPE in sAUD [13], but this could be attributed to the influence of divided attention or cognitive flexibility deficits frequently reported in this population on GPE evaluation. Our results confirm previous findings by demonstrating an impaired GPE in sAUD patients and further documenting significant visuoperceptive deficits in this population, as reported in various paradigms over the last few decades (see [6] for a review). Our observation of an absence of GPE thus enhances our understanding of visuoperceptive impairments in sAUD and emphasizes the need to consider these deficits on both clinical and experimental levels. Clinicians frequently overlook visuoperceptive deficits when proposing cognitive remediation for patients with sAUD, as they tend to focus on high-level cognitive functions. This approach ignores the fact that visuoperception and its associated anatomical/functional changes in brain area processing of visual stimuli play a crucial role in disease persistence and relapse, particularly through the disrupted connectivity between visual and frontal regions [29,30]. On the other hand, future research exploring the cognitive correlates of sAUD should consider visuoperception, as visual abilities underlie high-level cognitive functions. The deficits reported earlier for attention, memory or executive functions might thus partly result from visuoperceptive impairments rather than from high-level cognitive alterations alone. In view of the large-scale visuoperceptive impairments in sAUD, as further extended in the present study, we urge future research exploring cognitive abilities in this population to take visual abilities into consideration.

Thirdly, we conducted the first experimental exploration of GPE in KS. We identified a specific pattern where patients with KS, beyond the absence of the GPE, even presented a reverse performance, with local interference in six out of seven patients (see Figure S1, Supplementary Materials); they were slower to answer in the incongruent global than congruent global condition, which was not found in the local conditions. Such an atypical pattern indicating a local precedence effect has been reported in psychopathological states such as autism [31,32] or schizophrenia [33]. However, our results constitute the first report of such an effect in addictive disorders to our knowledge. Thus, we provide evidence of a gradient of deficits in alcohol-related disorders, from the absence of the classical GPE observed in healthy controls among patients with sAUD to a reverse local precedence effect in KS patients. Such a gradient supports the continuum hypothesis, which postulates a progressive worsening of cognitive and cerebral impairments during the transition from sAUD to KS [34]. It could also explain the discrepancies reported in earlier studies exploring GPE in sAUD, as the local precedence observed in some reports [14] might be related to the inclusion of patients presenting various levels of cognitive difficulties in a common experimental group, from patients with low/moderate cognitive impairments (potentially presenting a reduced or absent GPE, in line with our sAUD group) to patients with stronger deficits or even undiagnosed neurological complications (potentially presenting a local precedence effect, in line with our KS group). Moreover, it can be postulated that as the corpus callosum plays a critical role during global–local processes [14] and given the lateralization effect regarding the processing of local (left hemisphere) and global (right hemisphere) information [35,36], callosal abnormalities in KS participants [37,38] may lead to a lack of global–local integration by both hemispheres, generating a strong impairment during global–local processes compared to both control and sAUD participants. Callosal disconnection would impact the transfer of information and communication between the hemispheres, as noted in other clinical populations with callosal abnormalities [39]. Upcoming neuroscience studies should clarify the brain modifications underlying these GPE modifications and, more globally, the links between visuoperceptive abilities and brain impairments in alcohol-related disorders.

One could argue that our results are due to the presence of only women in the KS group of participants. Kimchi et al. [40] found that women and men are quite similar in their processing of global and local aspects of visual compound stimuli. Both women and men were faster during global processing than local processing, exhibiting the GPE typically observed in our control group of participants. Additionally, Kimchi et al. [41] found that women were more sensitive to global distractors than men. In the present experiment, we found a reverse pattern of results in the KS group of participants (i.e., local interference effect), even with only women in this group. This finding rules out the possible explanation that our results are due to the effect of gender rather than the impact of the disease. It is also important to note that gender differences are not always present in global/local studies using letter compound stimuli (see, for instance, [18], pages 209 and 210). Finally, patients with KS were older than patients with sAUD and HC. It has recently been shown that, compared to younger adults, older participants are disproportionately less efficient during local processing [40]. In the present study, the presence of a more pronounced local interference in KS participants compared to sAUD and HC participants cannot be explained by an age effect.

In conclusion, while our results are preliminary and need to be confirmed in larger groups with balanced sample sizes and matched for age and gender, as well as extended to other tasks testing visuoperceptive abilities, we have provided evidence of a gradient of GPE impairment in alcohol use disorders. Specifically, we have (a) replicated the classical GPE among healthy low-level drinkers, demonstrating the validity of our experimental design, (b) documented the lack of GPE among patients with sAUD, namely an absence of a significant difference in RT between local and global processing and (c) identified, for the first time, a reverse pattern in patients with KS, as they showed a local precedence effect. These findings emphasize the importance of considering local/global abilities among patients with alcohol use disorders, as these processes are related to crucial abilities ranging from basic object recognition to complex tasks such as driving or writing [42]. The impairments associated with such processes may therefore contribute to the development and maintenance of addictive disorders by promoting well-known traits of excessive alcohol consumption, such as impaired everyday activities, reduced well-being or increased negative emotions.

## Figures and Tables

**Table 1:** Demographic and clinical description of the sample.

	Healthy Controls N = 41	Patients with sAUD N = 16	Patients with KS N = 7
Age *	46.00 ± 11.91	47.40 ± 11.35	56.60 ± 4.65
Gender *	27 M/14 W	16 M/0 W	0 M/7 W
Education (in years)	11.40 ± 1.76	11.80 ± 2.51	10.70 ± 1.38
AUDIT score	<7 for men	26.38 ± 7.22	Not available
	<6 for women		
Daily alcohol consumption	<1	15.09 ± 6.74	Not available
Abstinence duration	-	7.38 ± 2.60	About 10 years ^1^
CVLT			
Trail 5	-	-	6.71 ± 2.89
Short-term free recall	-	-	1.43 ± 1.27
Long-term free recall	-	-	1.57 ± 1.72

Means ± standard deviation; AUDIT: Alcohol Use Disorder Identification Test; KS: Korsakoff’s syndrome; sAUD: severe Alcohol Use Disorder; M: men; W: women; CVLT: California Verbal Learning Test; * significant difference between groups; ^1^ except for one patient with KS who had been abstinent for less than one year.

## Data Availability

All data and materials used within this study will be made available, upon reasonable request, to research groups wishing to reproduce/confirm our results.

## Supplementary Materials

The following supporting information can be downloaded at: https://www.mdpi.com/article/10.3390/jcm12113655/s1, Figure S1: Individual mean RTs for each KS Patient. All patients present a pattern that corresponds to the effects described in the manuscript, except for KS7.
