# Supplementary material for: Impaired Global Precedence Effect in Severe Alcohol Use Disorder and Korsakoff’s Syndrome: A Pilot Exploration through a Global/Local Visual Paradigm
Source: J Clin Med. 2023 May 25;12(11):3655. doi: 10.3390/jcm12113655 (PMC10253928; doi:10.3390/jcm12113655)

**Supplementary materials Figure S1: Individual mean RTs for each KS Patient.**

All patients present a pattern that corresponds to the effects described in the manuscript, except for KS7.


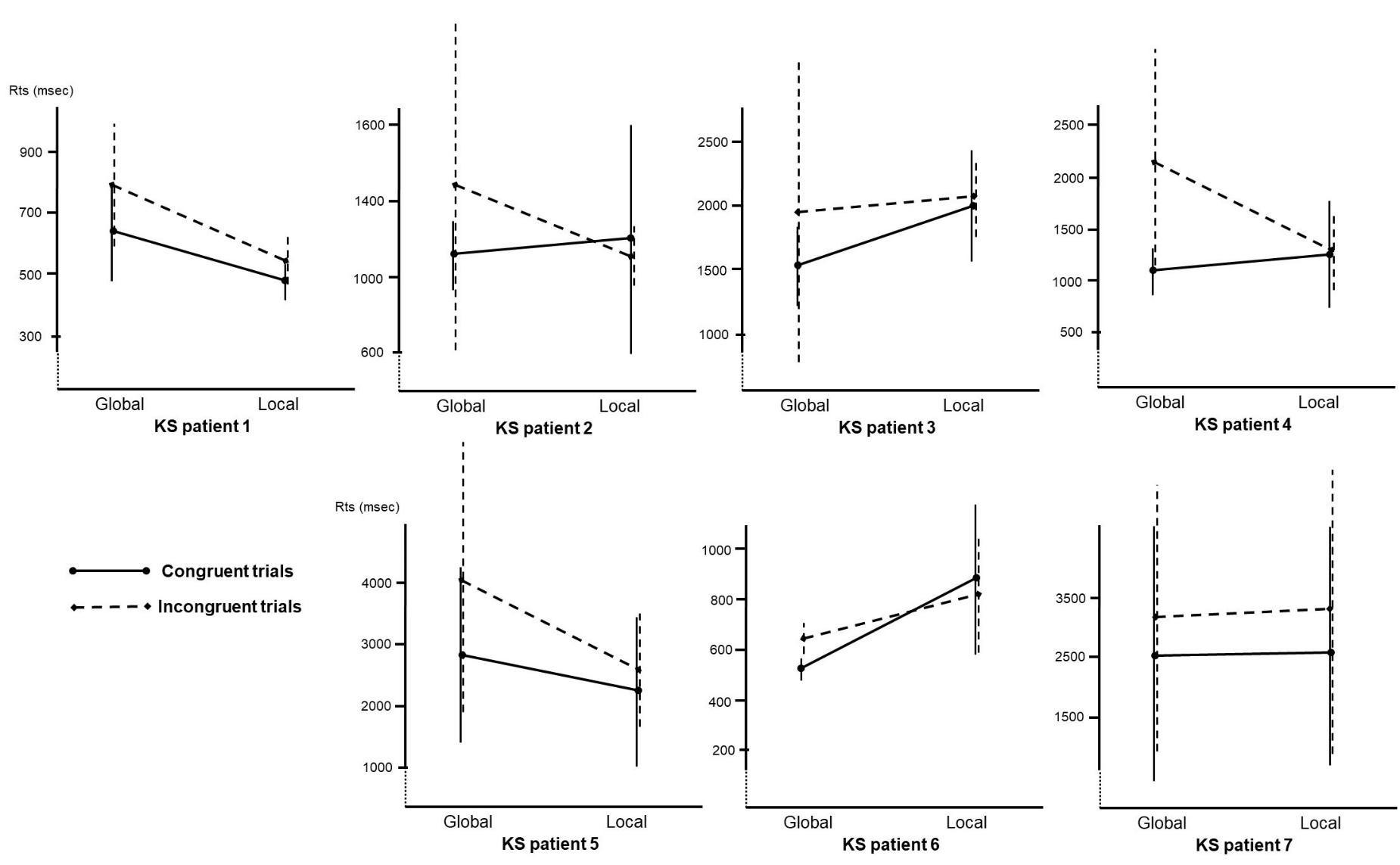

Supplement: Supplementary file 1 [file jcm-12-03655-s001.zip › jcm-2312796-supplementary.docx]
